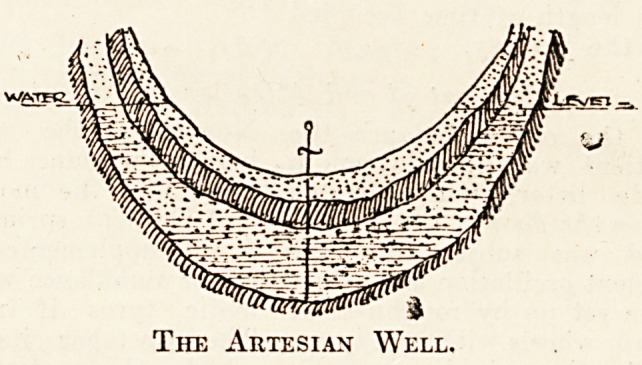# Hospital Architecture and Construction

**Published:** 1911-02-25

**Authors:** 


### Hospital Architecture and Construction.

[Communications on this subject should be marked "Architecture" in the left-hand top corner of the envelope.]

### THE WATER-SUPPLY OF HOSPITALS.

### I. TYPES OF WELLS.

In Cottage Hospitals and Sanatoria situate in the outlying parts of this country and our dominions oversea the problem of water supply is one which often presents difficulty to the management. How and where is a well to be sunk ? What quantity of water will be required to keep the supply sufficient for all possible contingencies, and what are the best methods of storage ? Of wells there are three kinds, namely, the shallow well, the deep well, and the artesian, the last-named being really a type of deep well.

The following sketches will show clearly how these appellations are arrived at :

The Shallow Well.

It will be seen that the shallow type of well is that in which the water percolates through the ground until it comes to a type of soil or rock through which it cannot pass and there it stands until it reaches the level of the lowest lip, which is generally the outcrop of the impervious strata from whence it emerges as spring water.

The level of the plane of saturation is very variable, being rapidly affected by the rainfall, and as the depth of soil through which the water has to pass is usually comparatively small, shallow wells are at best doubtful sources of supply, and when

The Deep Well.

resorted to great care should be taken that no contaminating substances are allowed on the supply area, since rain-water, on account of its solvent nature, readily takes up and retains any impurities, particularly those of an organic nature. It was probably with this in mind that the Rivers Pollution Commission labelled shallow wells as dangerous.

The deep well is that kind which is now being sunk by many enterprising private firms and companies having offices, hotels, etc., in London, in order to effect an economy on the charges of the Metropolitan Water Board. It will be seen from the illustration that the boring goes through one or more impervious beds to a porous or water-bearing stratum underneath, which, in its turn, is again held by an impervious layer in a manner similar to that described in the shallow well. The terms "shallow" and "deep" it will therefore be seen, do not necessarily refer to the actual depth of boring required; in fact a shallow type of well will often be found actually deeper than a "deep" well. Deep wells are excellent sources of supply for domestic purposes; the rainfall which feeds them has percolated a considerable distance through the ground, and has thus become purified and aerated. Moreover it contains an amount of mineral matter in solution which it has collected in its passage, thus removing the soft character inseparable from ordinary rain-water. Deep wells are not so readily affected by varying rainfalls, and are therefore more reliable; the level of water standing in a deep well depends on the perfection of the retaining basin, the elevations of the retaining beds, and the line of outflow; the principle being the same as that of a shallow well. There is a continuous flow of water in saturated strata from the collecting area towards the outlet, which may be the bed of the river, the shore of a lake, or the sea. The surface level of this moving

The Artesian Well.

body of water depends upon the structure of the beds through which it flows.

Should the point selected for sinking a deep well be so situated that owing to undulations in the strata the upper surface of the water layer lies beneath the virtual water-level in the saturated beds, then the water, were it not for the resistance of the air, would rise to the height of the virtual water-line opposite the point. This constitutes what is known as an artesian well, the name being derived from a French province where this type of well was developed; it will thus be apparent that, as before stated, the artesian is only a particular condition of the deep well.

## Figures and Tables

**Figure f1:**
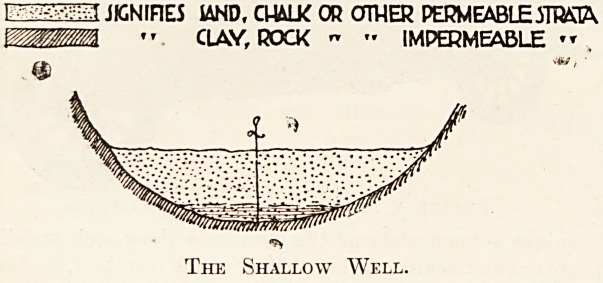


**Figure f2:**
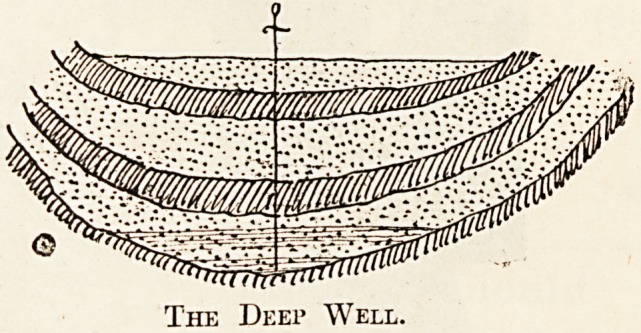


**Figure f3:**